# Supplementary material for: STING-Activating Polymer–Drug Conjugates for Cancer Immunotherapy
Source: ACS Cent Sci. 2024 Aug 20;10(9):1765–81. doi: 10.1021/acscentsci.4c00579 (PMC11428287; doi:10.1021/acscentsci.4c00579)
Supplement: Supplementary file 1 — oc4c00579_si_001.pdf [file oc4c00579_si_001.pdf]

Supplemental Information

STING-Activating Polymer-Drug Conjugates for Cancer Immunotherapy

Taylor L. Sheehy[1], Alexander J. Kwiatkowski[2], Karan Arora[2], Blaise R. Kimmel[2,#], Jacob A. Schulman[1], Katherine N. Gibson-Corley[3], John T. Wilson[1-7,*]

[1]Department of Biomedical Engineering, Vanderbilt University, Nashville, TN 37232, USA.
[2]Department of Chemical and Biomolecular Engineering, Vanderbilt University, Nashville, TN 37232, USA.
[3]Department of Pathology, Microbiology, and Immunology, Vanderbilt University Medical Center, Nashville, TN 37232, USA.
[4]Vanderbilt Ingram Cancer Center, Vanderbilt University Medical Center, Nashville, TN 37232, USA.
[5]Vanderbilt Institute of Chemical Biology, Vanderbilt University, Nashville, TN 37232, USA.
[6]Vanderbilt Institute for Infection, Immunology and Inflammation, Vanderbilt University Medical Center, Nashville, TN 37232, USA.
[7]Vanderbilt Center for Immunobiology, Vanderbilt University Medical Center, Nashville, TN 37232, USA.

[#]current address: Department of Chemical and Biomolecular Engineering, The Ohio State University, Columbus, OH, 43210, USA
*Corresponding author: john.t.wilson@vanderbilt.edu

Supplemental Data:

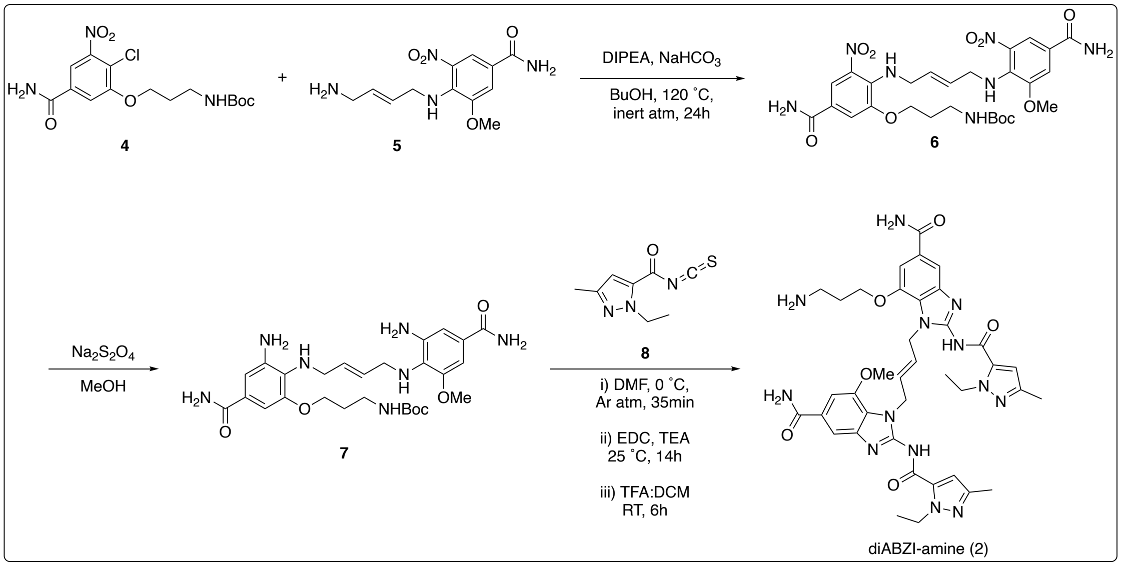

Scheme S1: Synthesis of diABZI-Amine

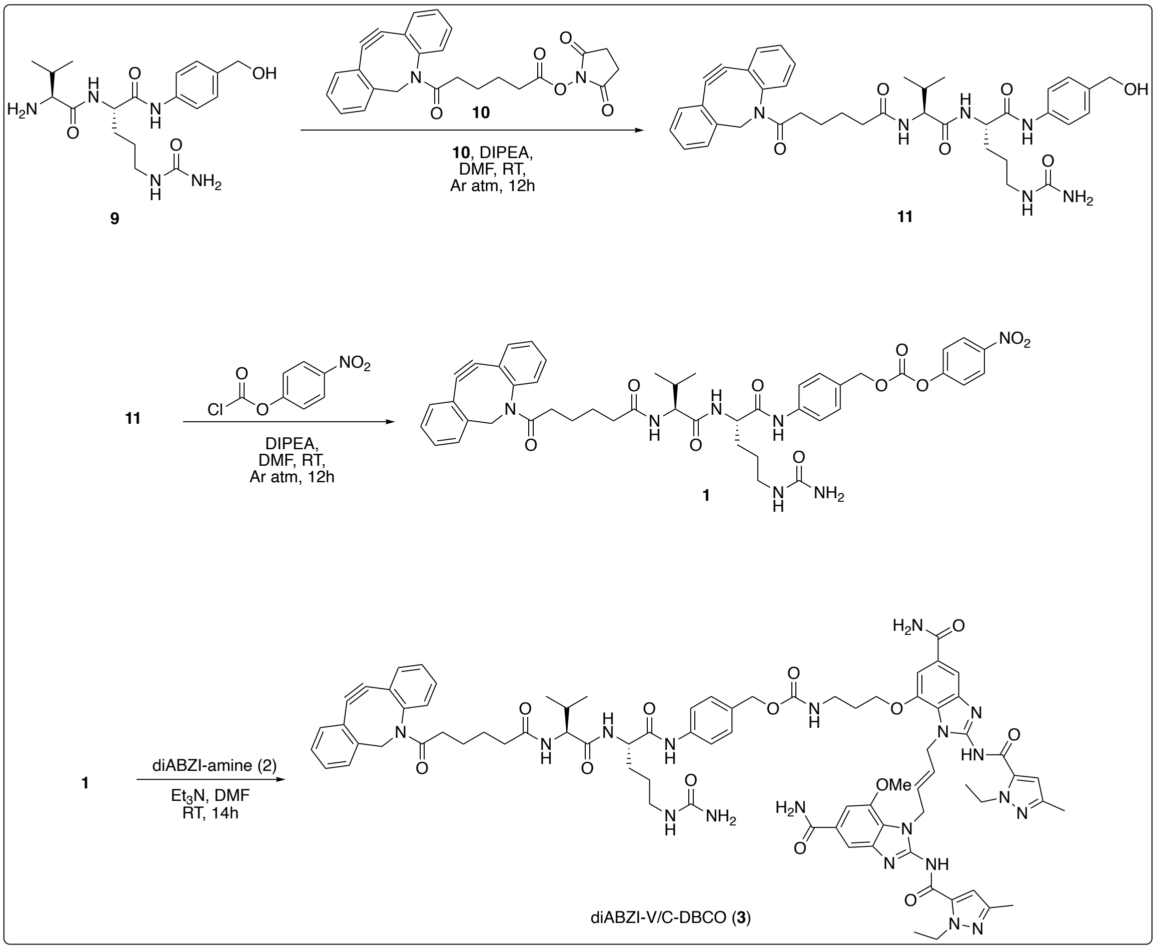

Scheme S2: Synthesis of diABZI-V/C-DBCO

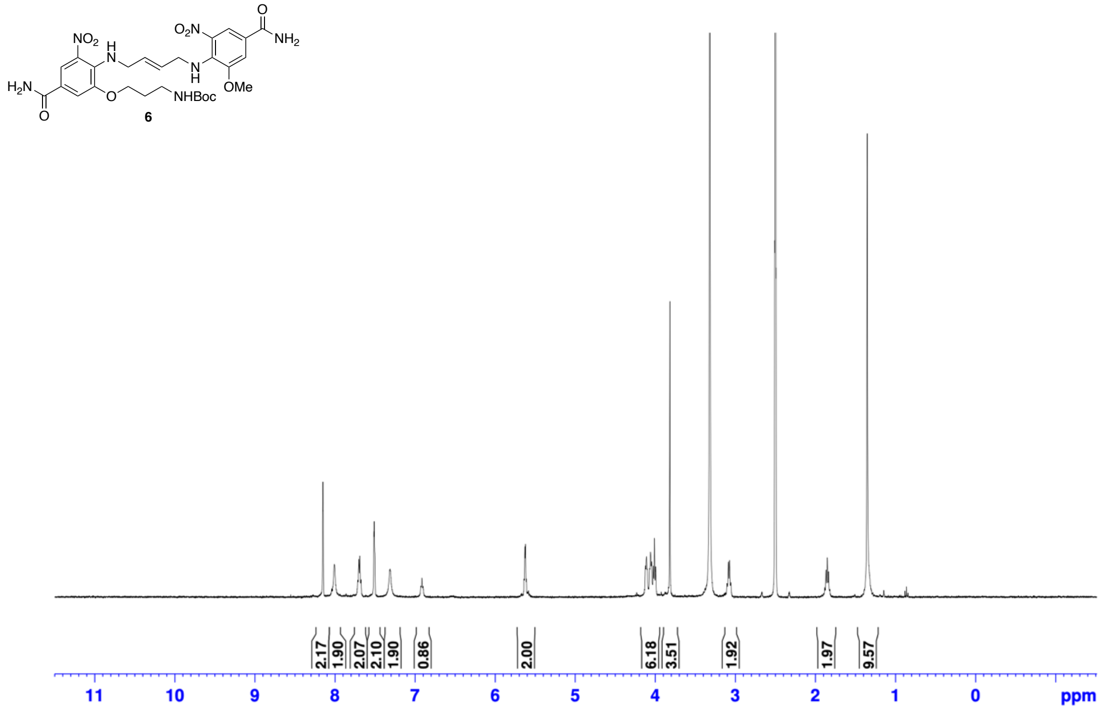

Figure S1: ^{1}H NMR of Compound **6**

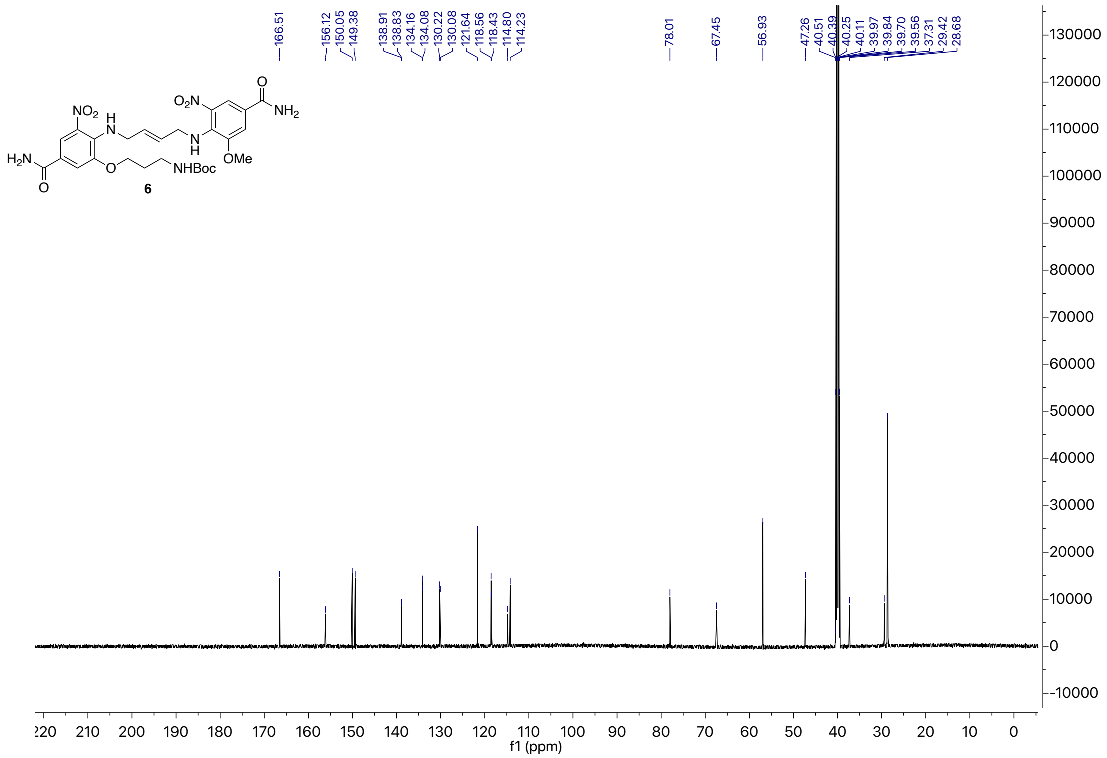

Figure S2: ^{13}C NMR of Compound **6**

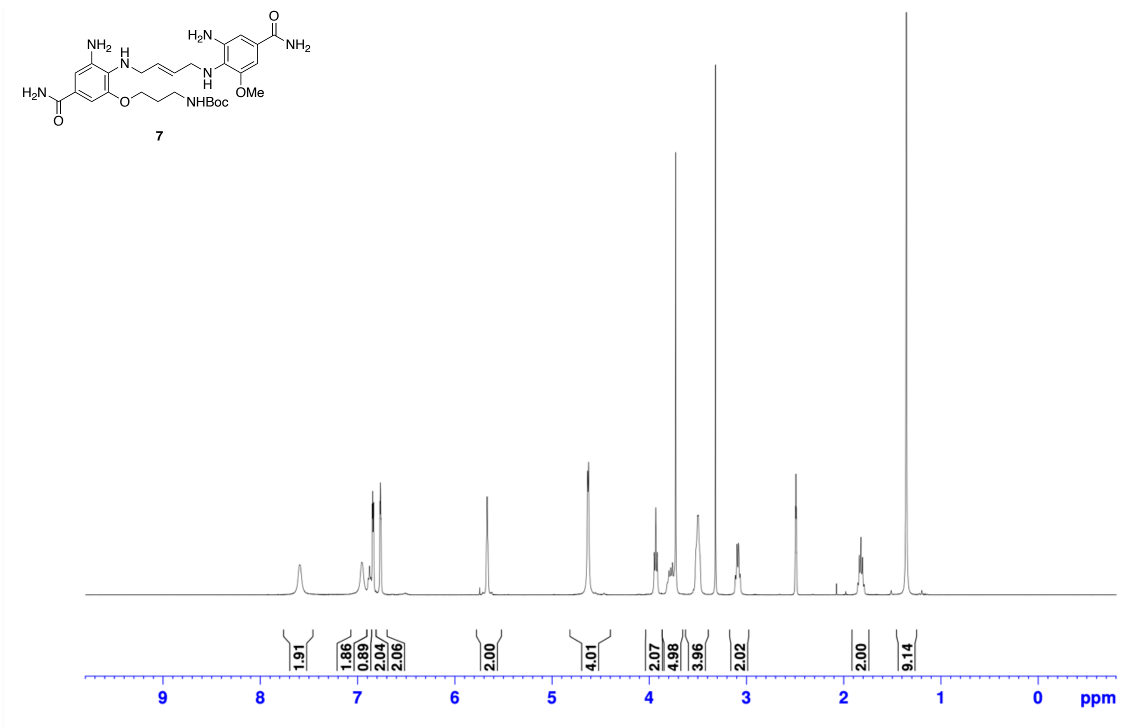

Figure S3: ^{1}H NMR of Compound **7**

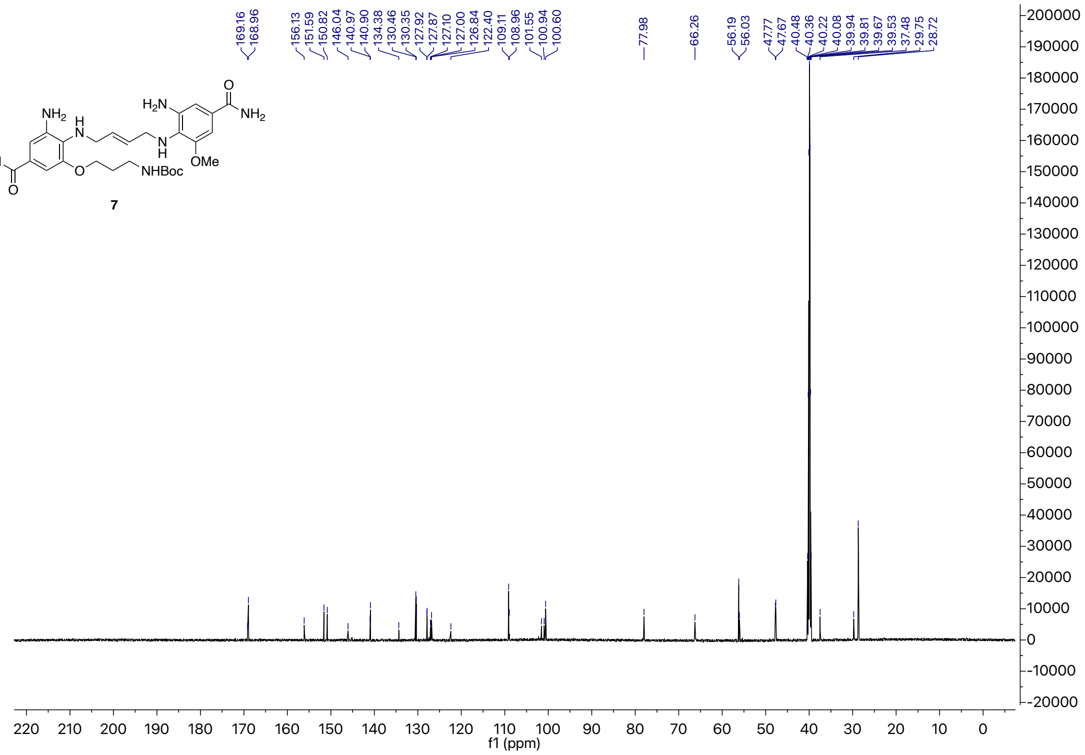

Figure S4: ^{13}C NMR of Compound **7**

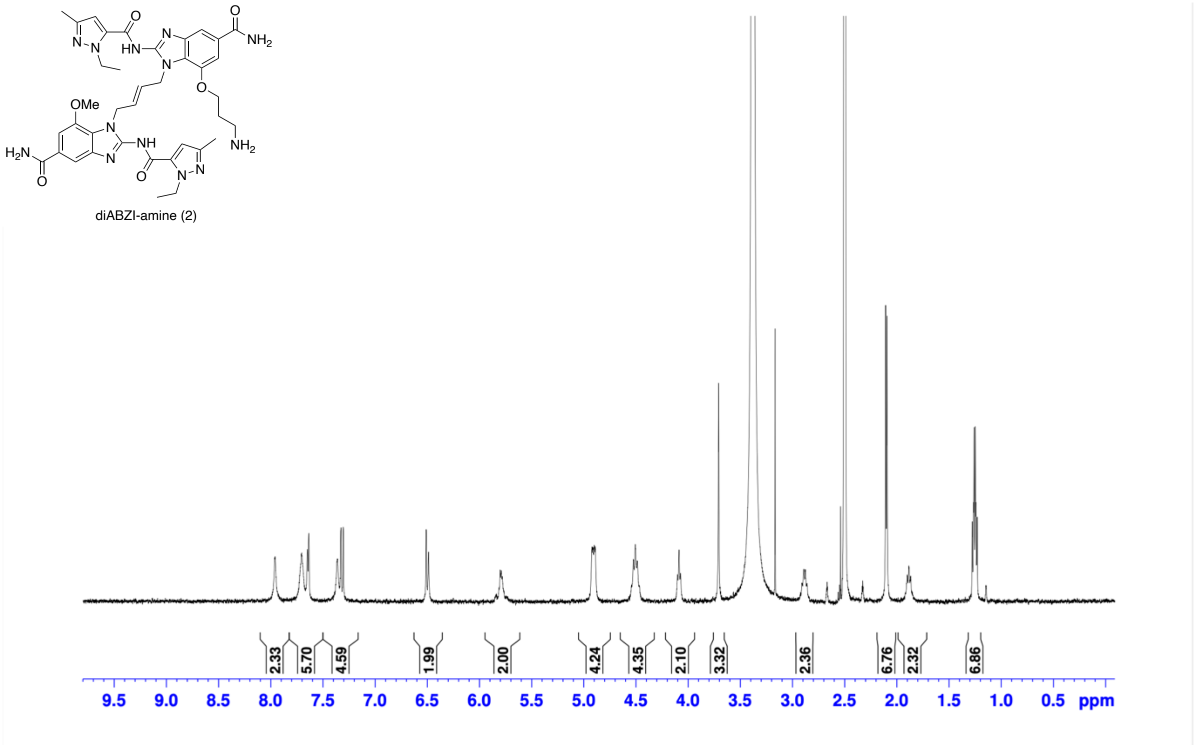

Figure S5: H[1] NMR of diABZI-Amine (2)

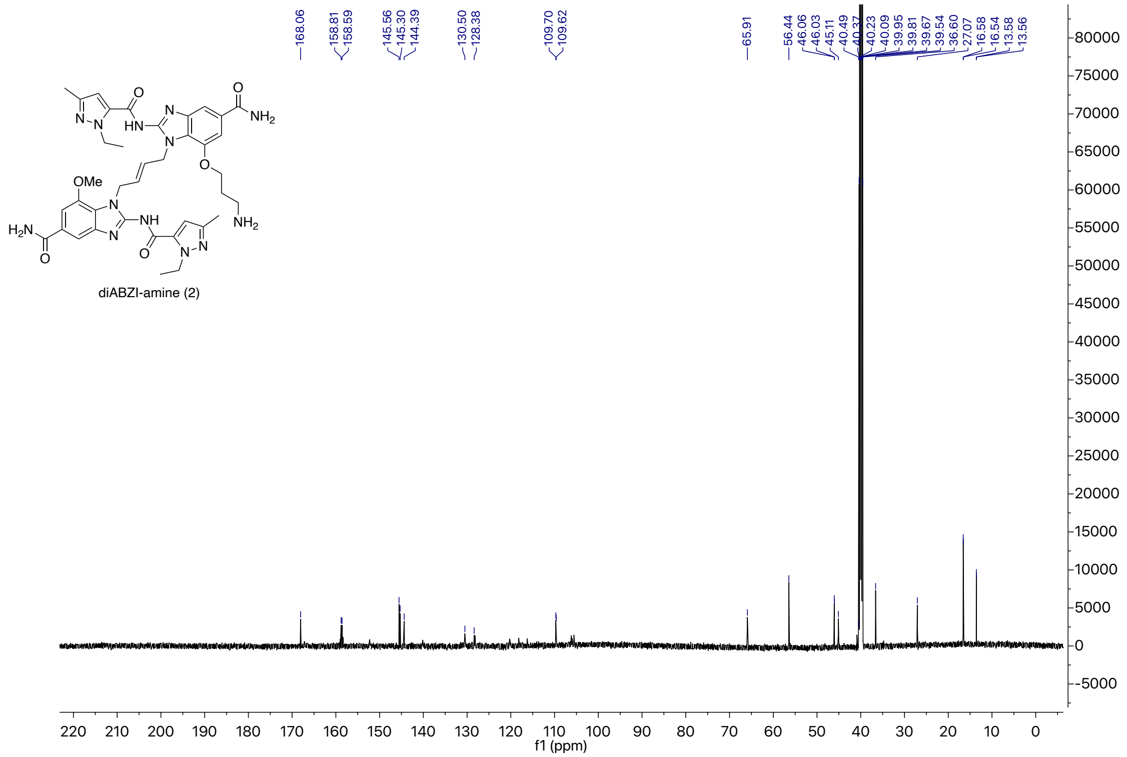

Figure S6: ^{13}C NMR of diABZI-Amine (2)

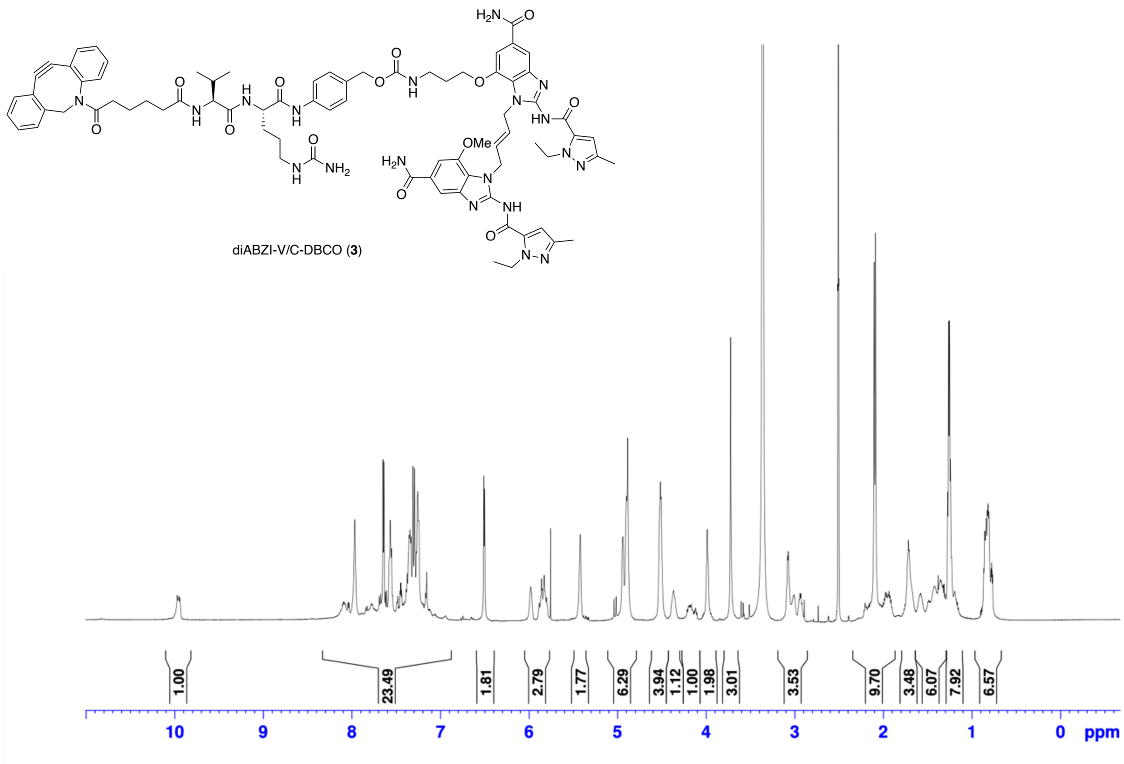

Figure S7: H^1 NMR of diABZI-V/C-DBCO (3)

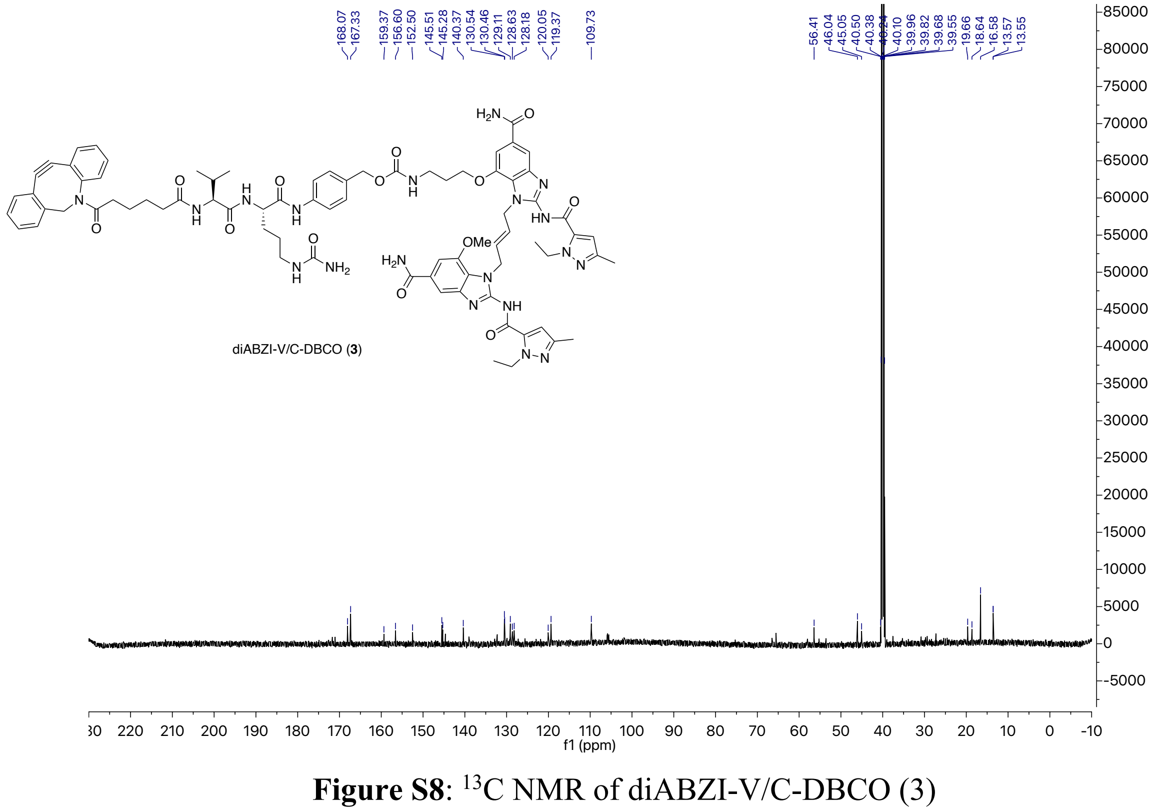

Figure S8: ^{13}C NMR of diABZI-V/C-DBCO (3)

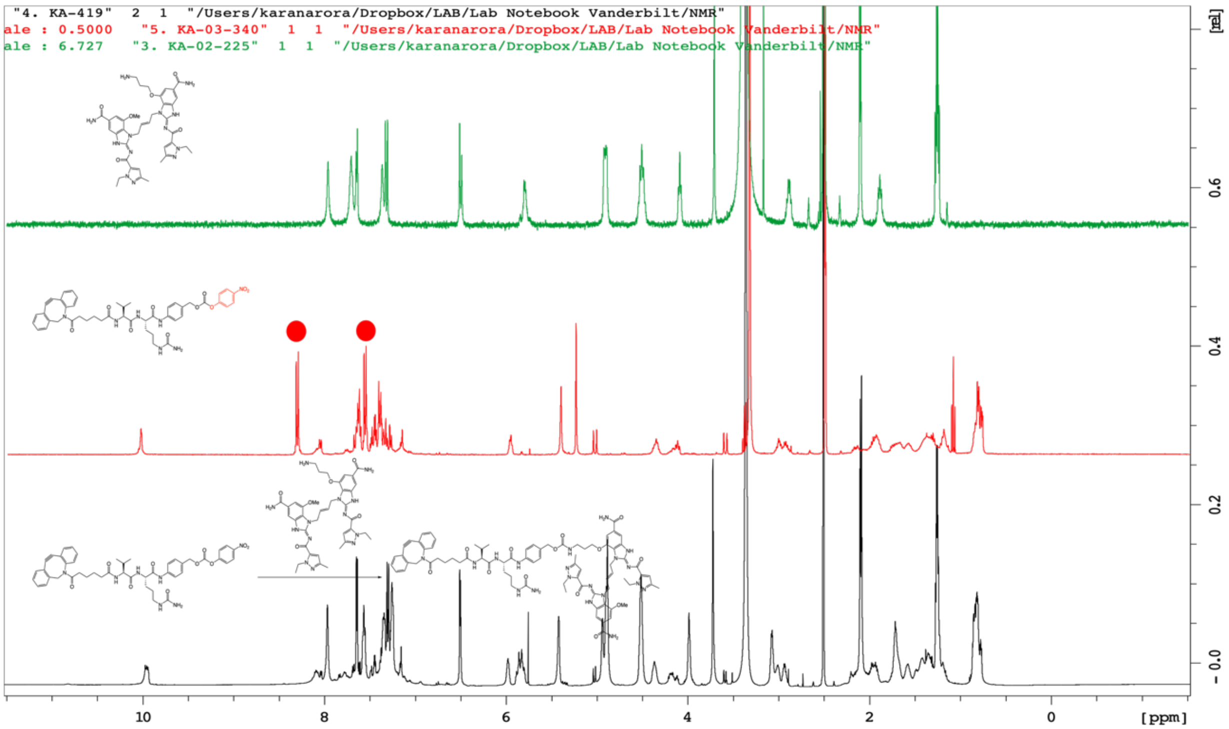

Figure S9: Summary of ^{1}H NMR for Synthesis of diABZI-V/C-DBCO

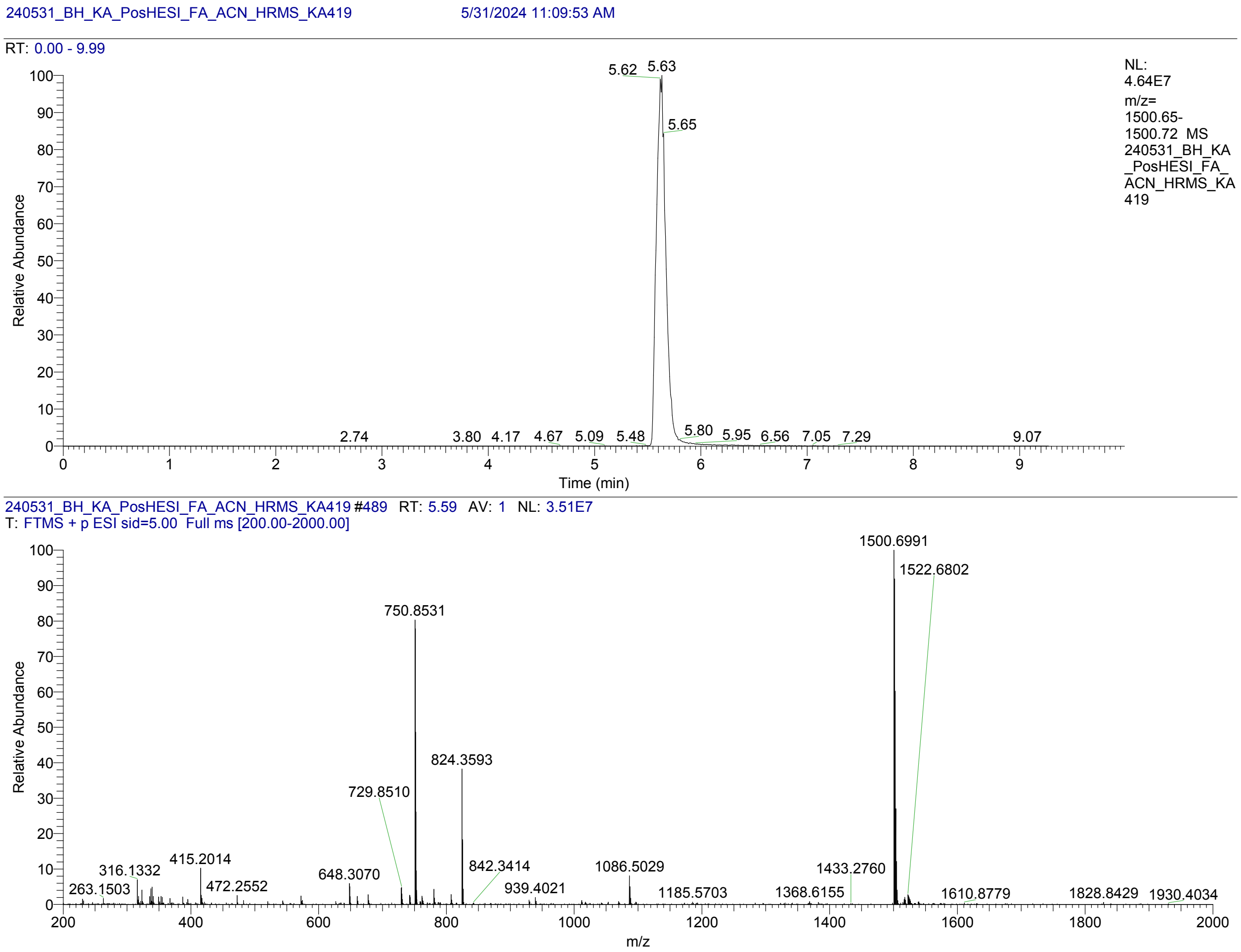

Figure S10: ESMS of diABZI-V/C-DBCO

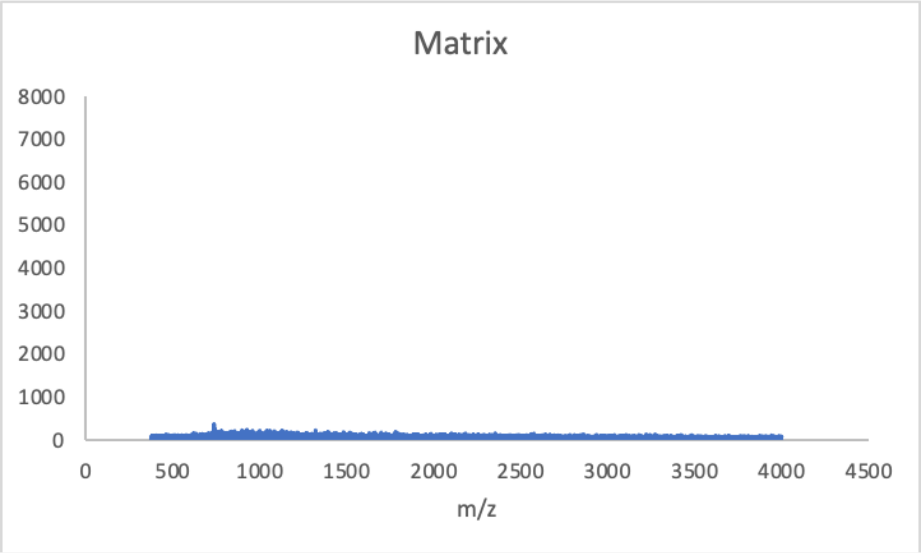

Figure S11: MALDI spectrum of matrix

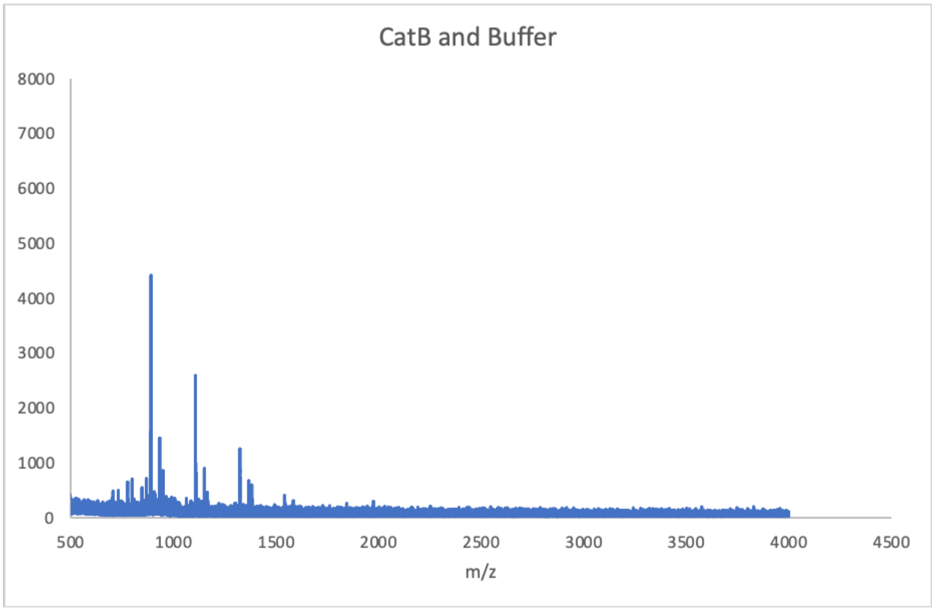

Figure S12: MALDI Spectrum of Cathepsin B and Buffer

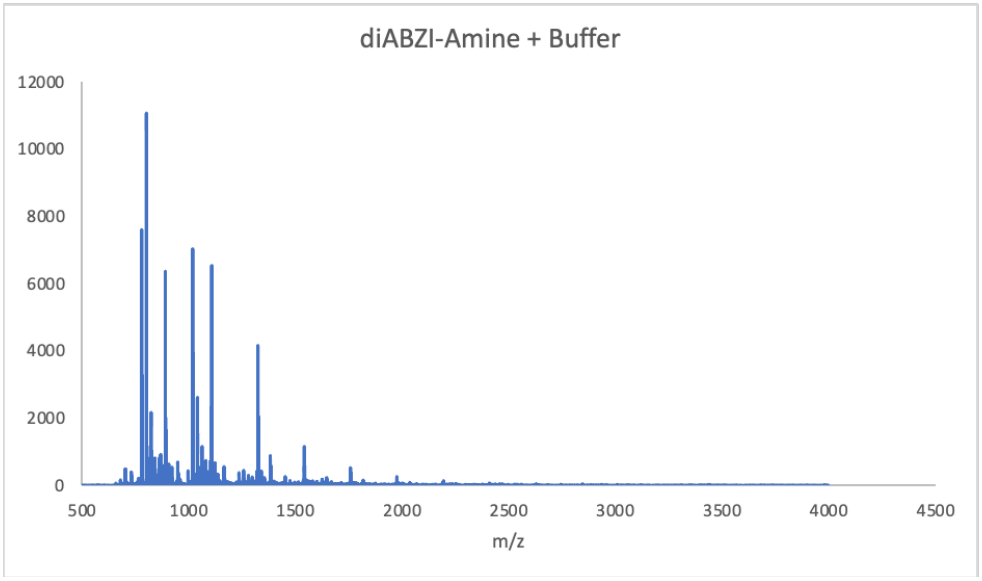

Figure S13: MALDI spectrum of diABZI-Amine and buffer

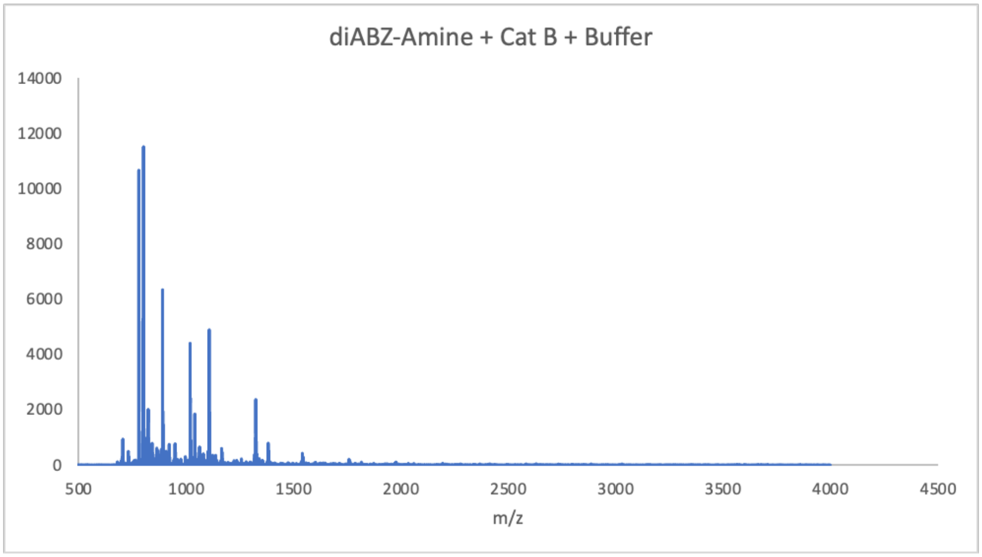

Figure S14: MALDI spectrum of diABZI-amine, cathepsin B, and buffer

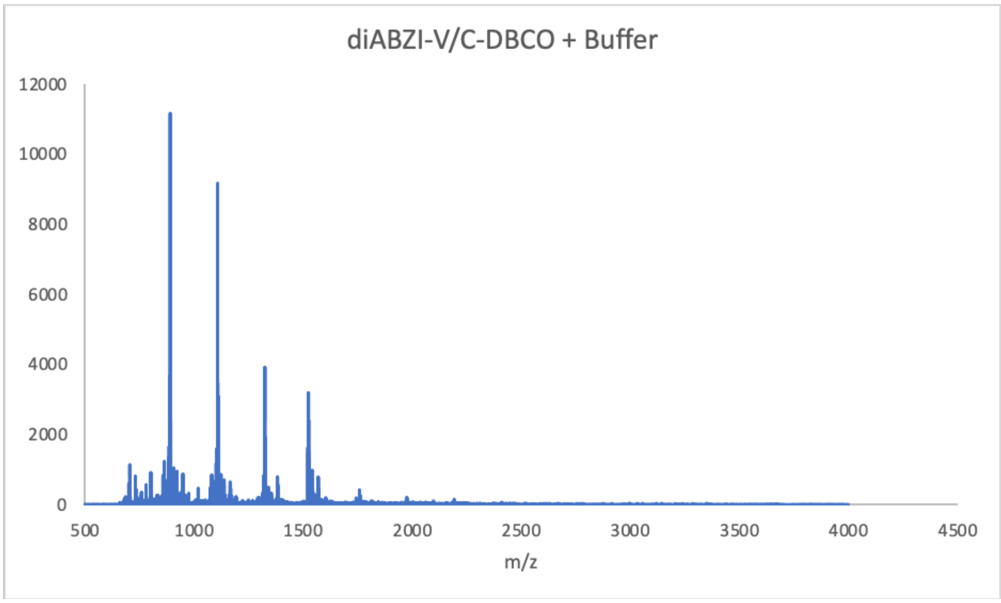

Figure S15: MALDI spectrum of diABZI-V/C-DBCO and buffer

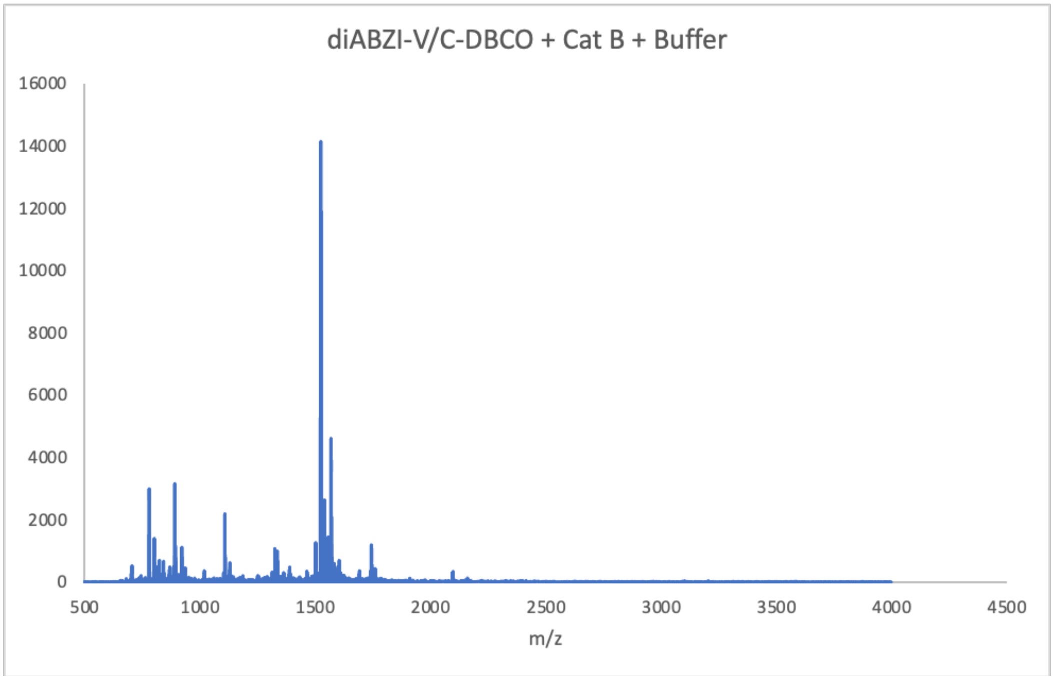

Figure S16: MALDI Spectrum of diABZI-V/C-DBCO, cathepsin B, and buffer

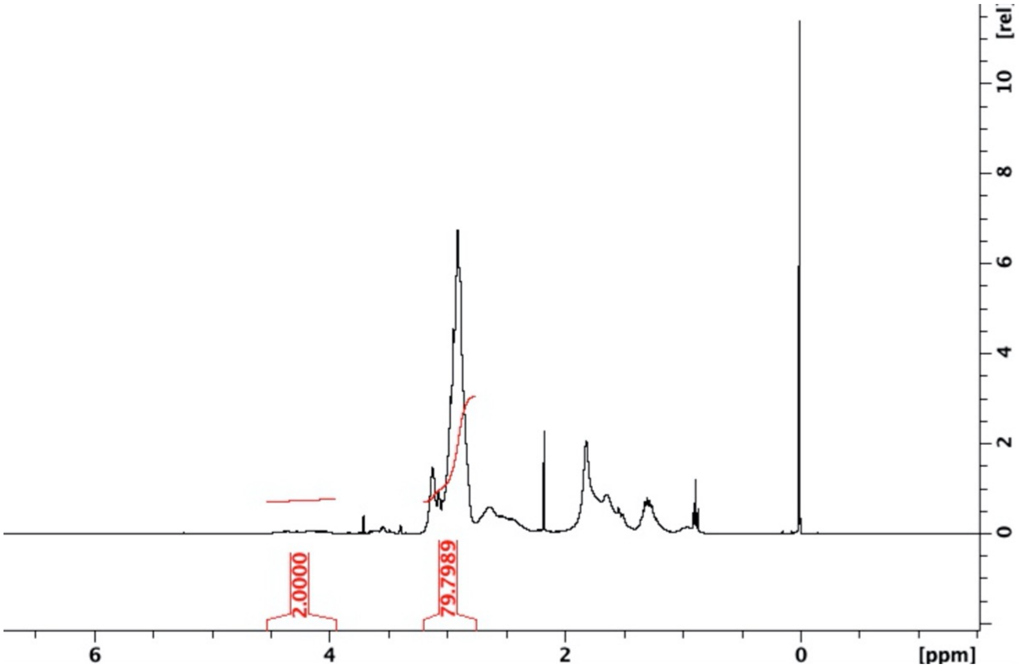

Figure S17: ^{1}H NMR of 25kDa DMA-*co*-AzEMA

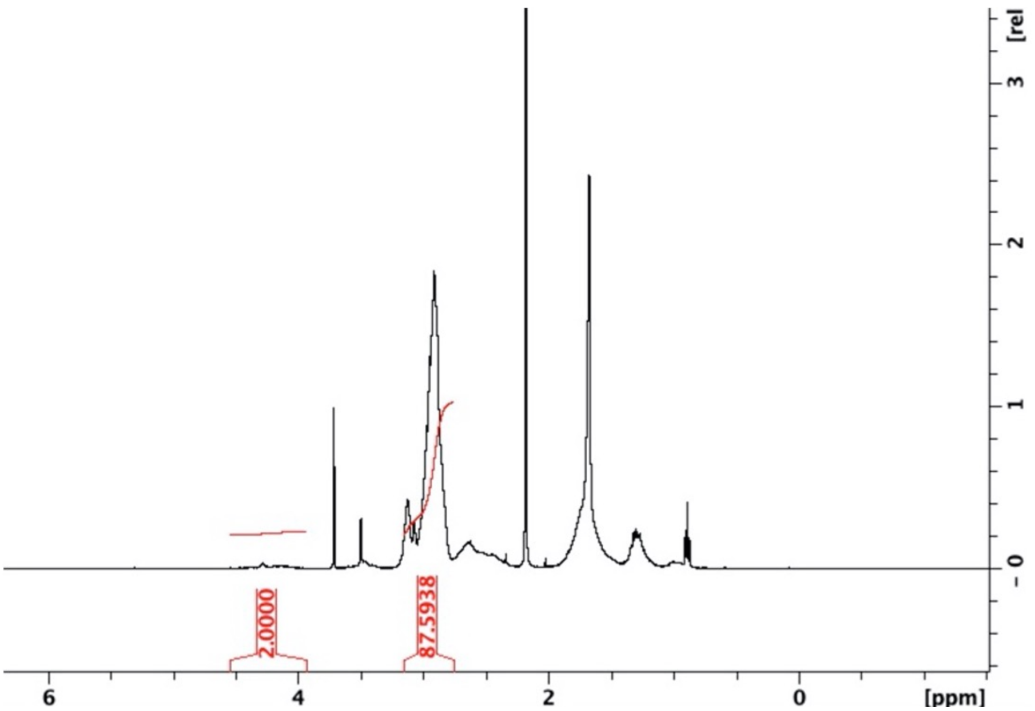

Figure S18: ¹H NMR of 100kDa DMA-*co*-AzEMA

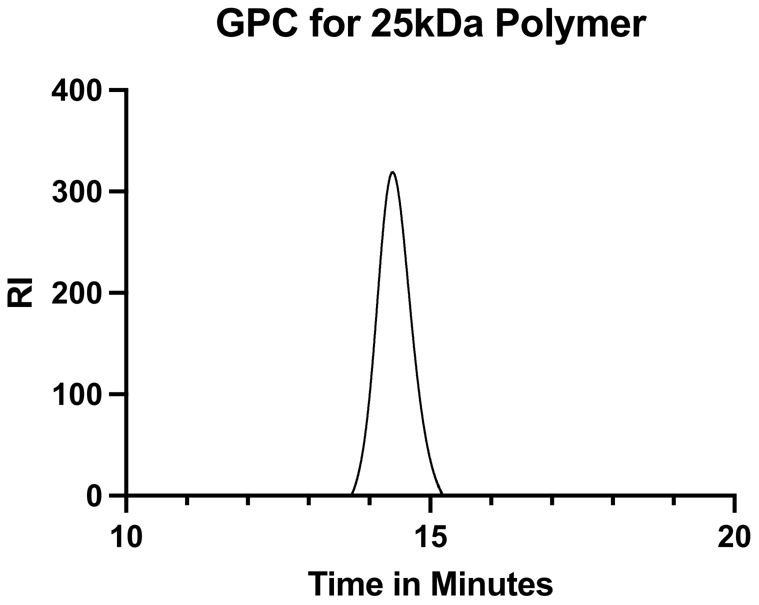

Figure S19: GPC trace of 25kDa DMA-*co*-AzEMA

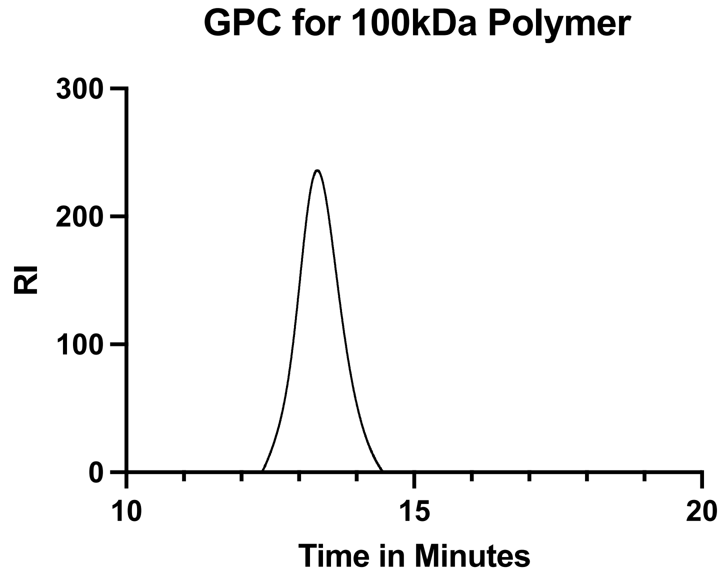

Figure S20: GPC trace of 100kDa DMA-*co*-AzEMA

Polymer	Mw NMR	Mw GPC	Mn GPC	PDI	Azides/chain (approximate)
25kDa	25,200	28,400	27,500	1.03	19
100kDa	101,000	132,000	116,400	1.13	72

Table S1: Summary of Polymer Properties

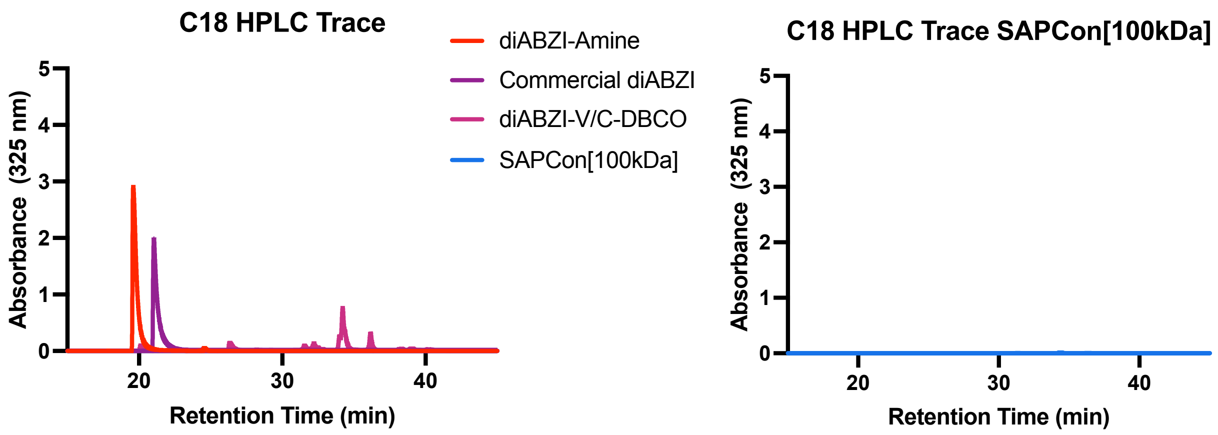

Figure S21: HPLC of free diABZI agonists and SAPCon[100kDa] demonstrating the absence of free diABZI in the purified conjugate. This HPLC method utilizes a C18 column in water with 0.1% TFA and acetonitrile with 0.1% TFA. The method starts with 85% water and 15% ACN for 5 minutes with a gradual decrease in water at -1% water/min for 40 minutes and then an increase in water at +8% water/min for 5 minutes.

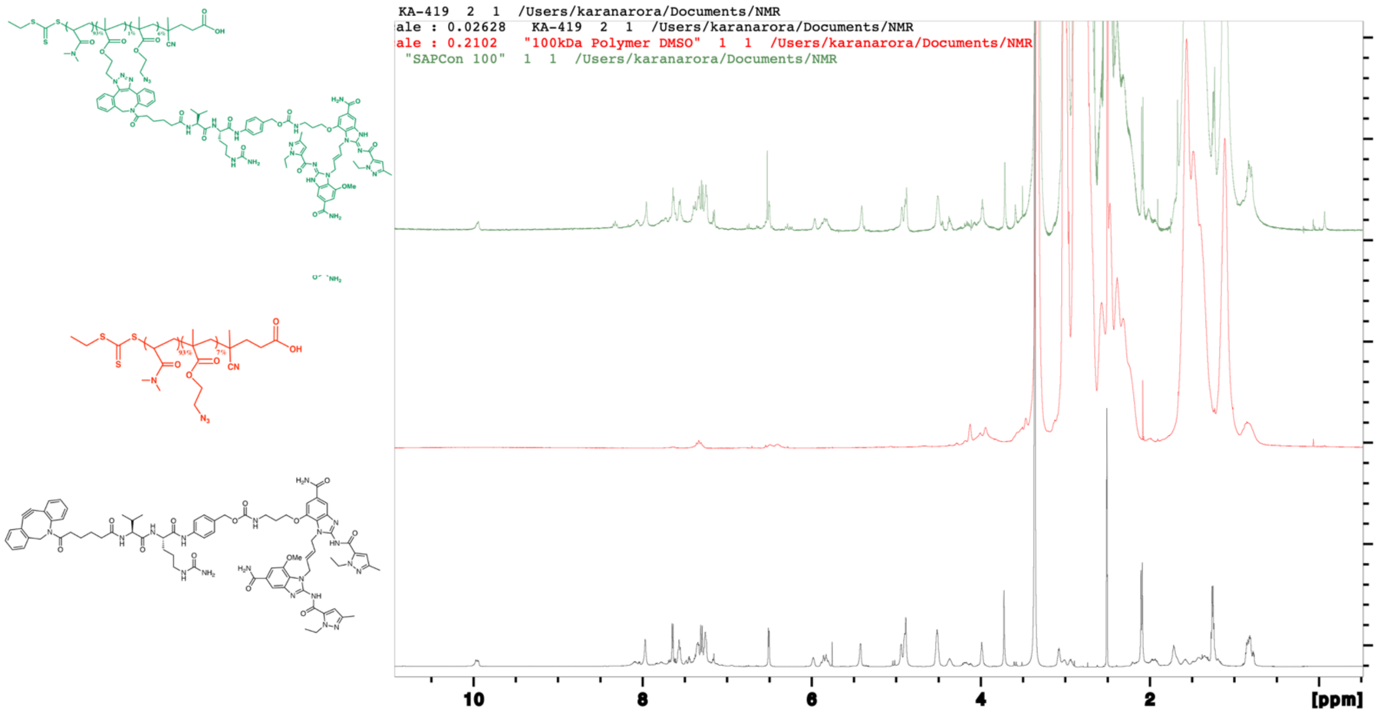

Figure S22: ¹H NMR of SAPCon[100kDa] (top) compared to and 100kDa DMA-*co*-AzEMA free polymer (middle) diABZI-V/C-DBCO (bottom).

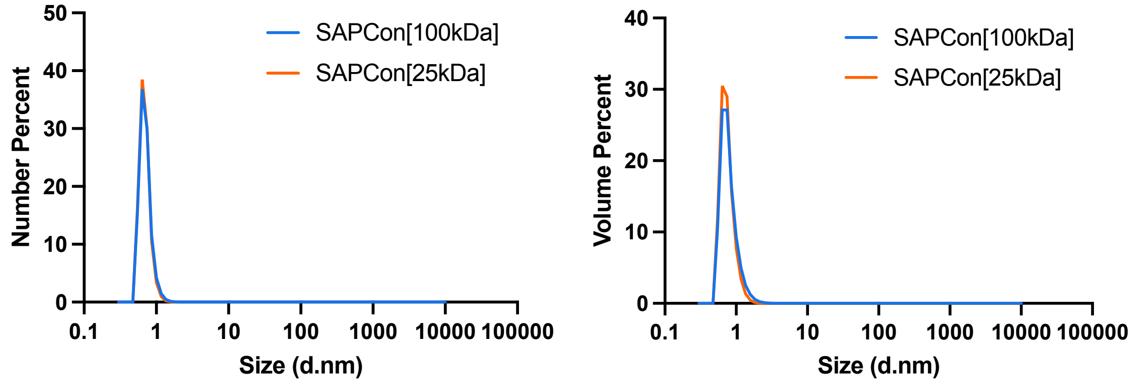

Figure S23: Dynamic light scattering of SAPCon[25kDa] and SAPCon[100kDa]

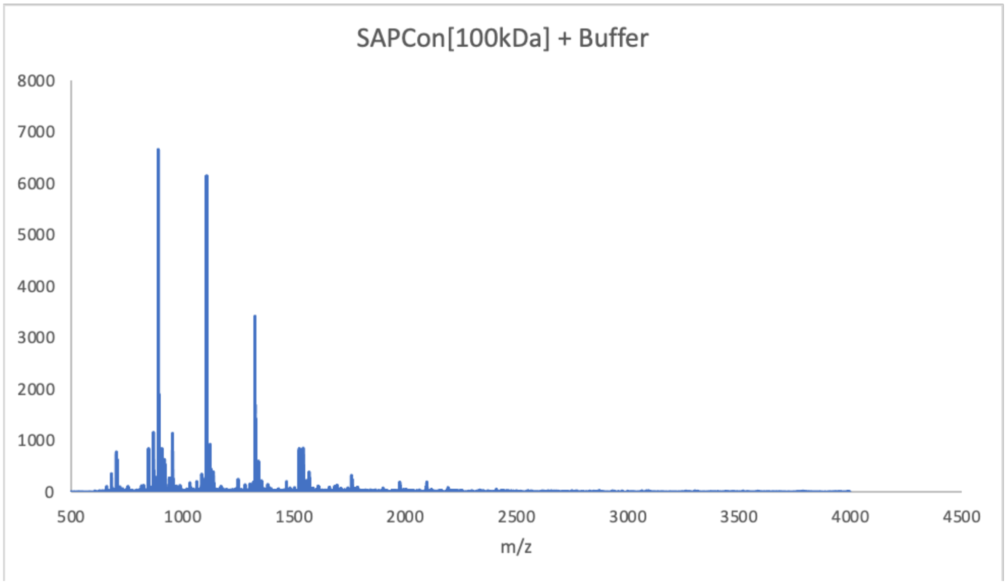

Figure S24: MALDI spectrum of SAPCon[100kDa] and buffer

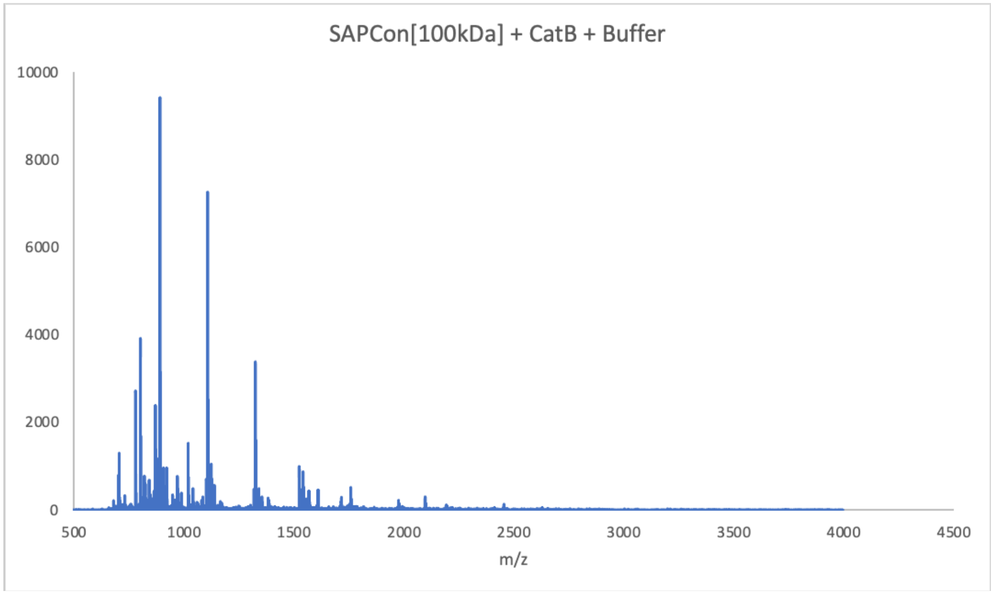

Figure S25: MALDI Spectrum of SAPCon[100kDa], cathepsin B, and buffer

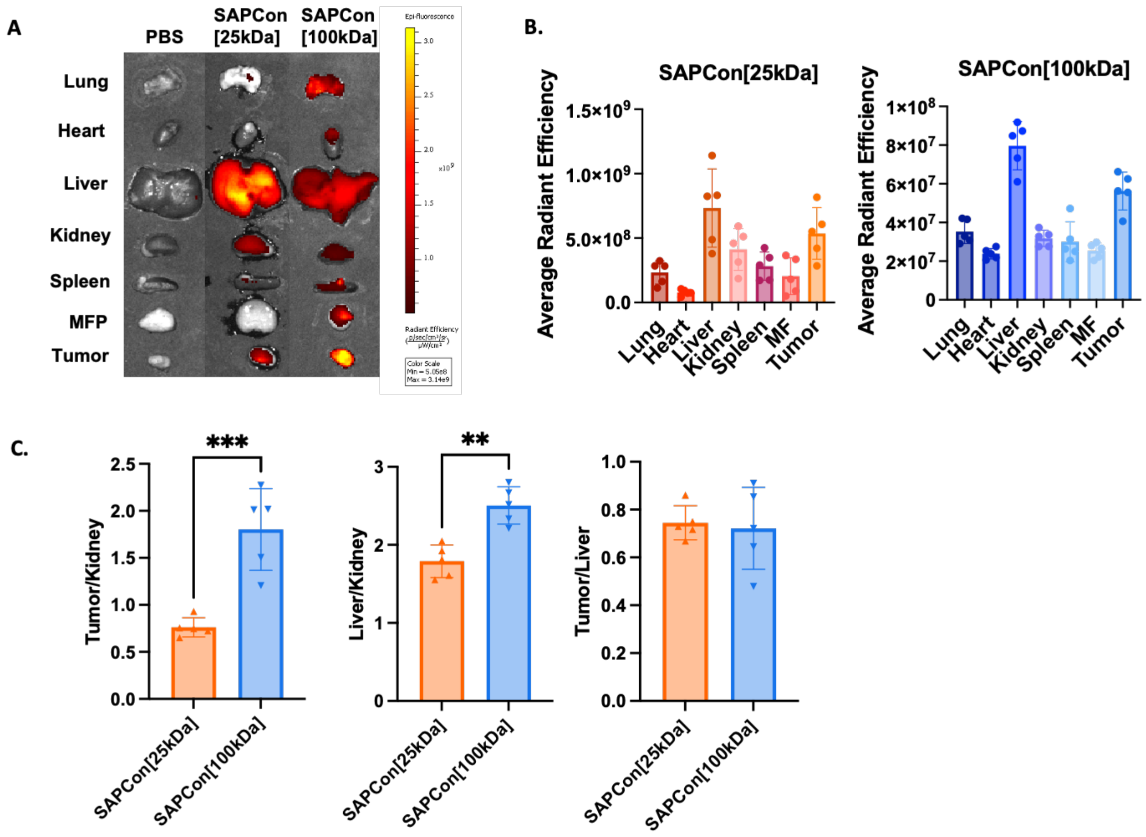

Figure S26: Biodistribution at 24 hours. **A)** Representative IVIS fluorescent images of excised EO771 tumors and organs 24 h following administration of Cy5-labeled SAPCon. **B)** Quantification of tissue fluorescence measured with IVIS imaging 24 h following administration of Cy5-labeled SAPCon (n=5). **D)** Ratios of tissue fluorescence between tumor and/or organs.

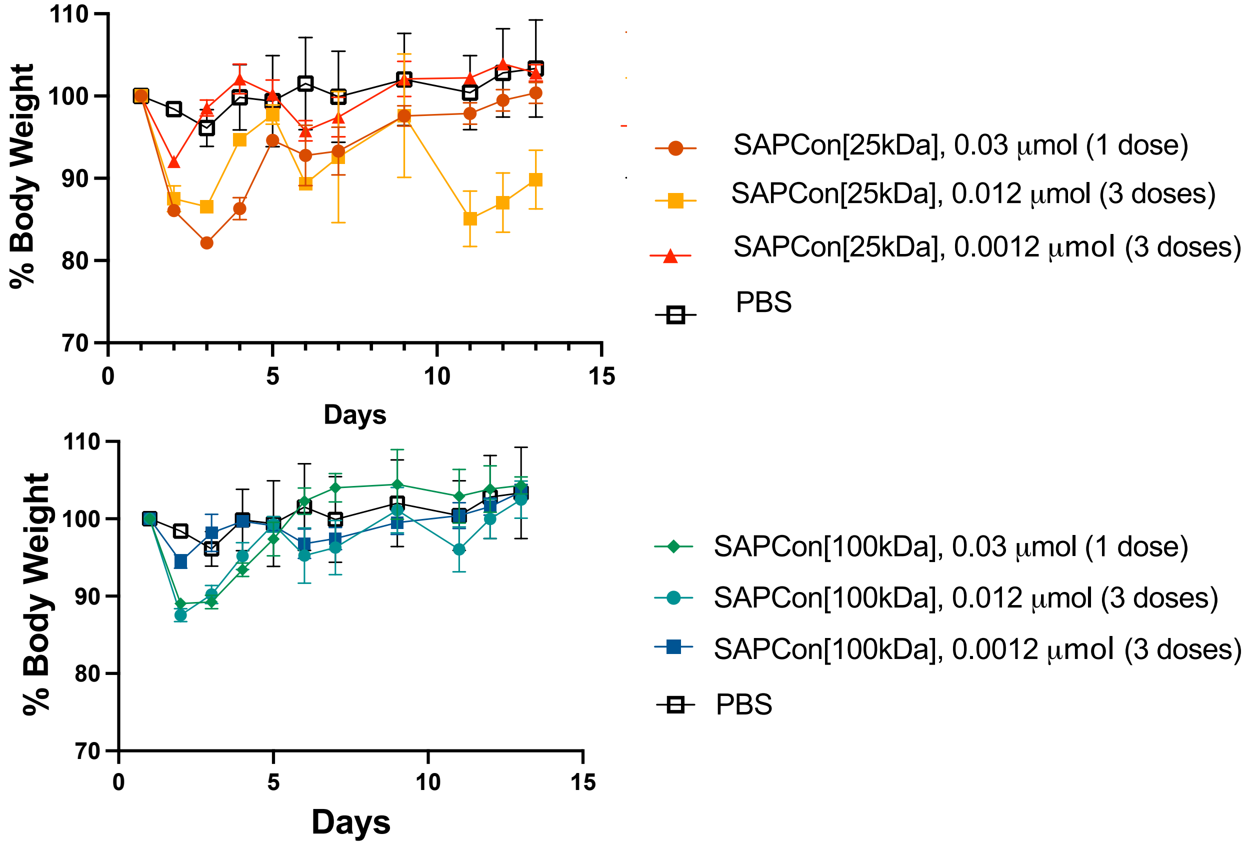

Figure S27: Transient weight loss in dose-finding toxicity study for SAPCon[25kDa] and SAPCon[100kDa] in healthy (n=13) C57BL/6 mice.

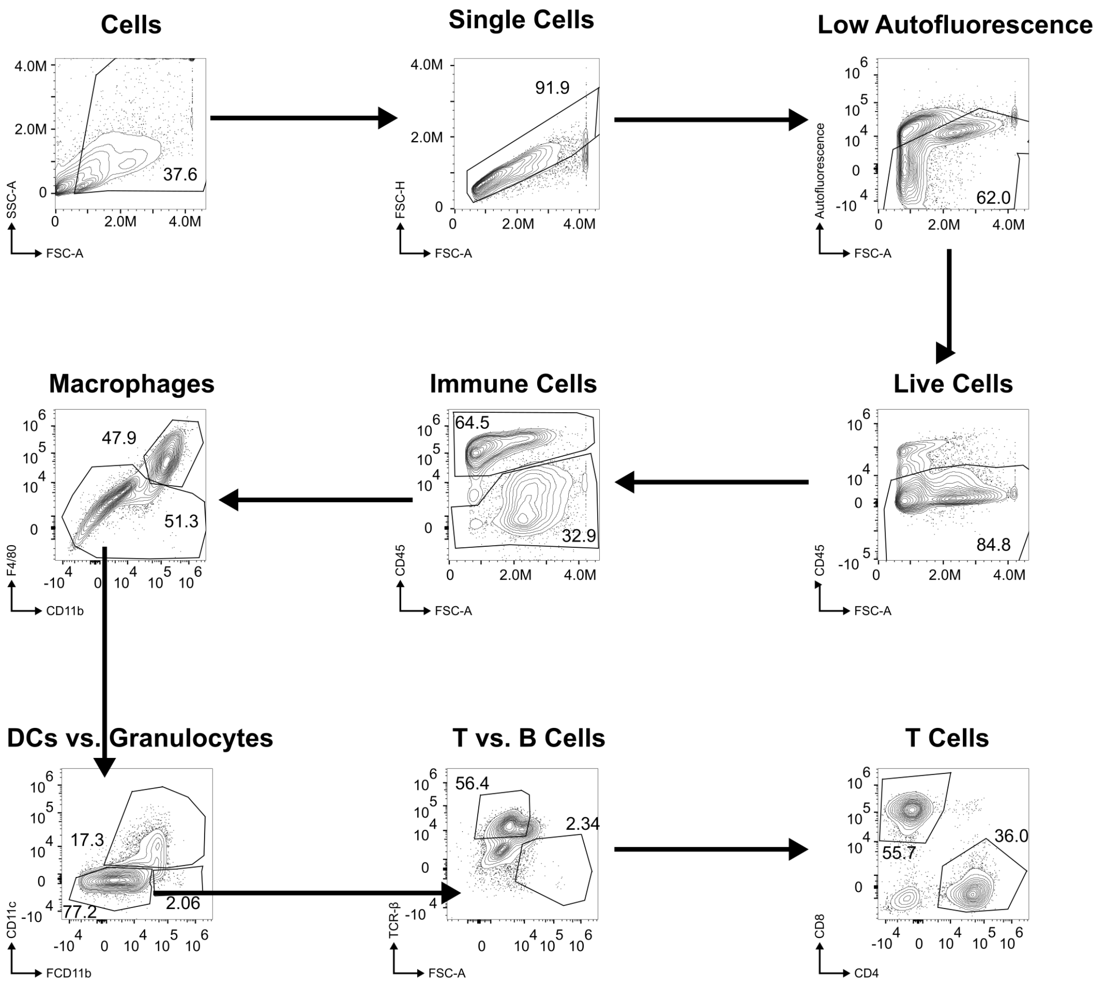

Figure S28: Flow cytometry gating for cell identification in Cy5 tumor uptake panel

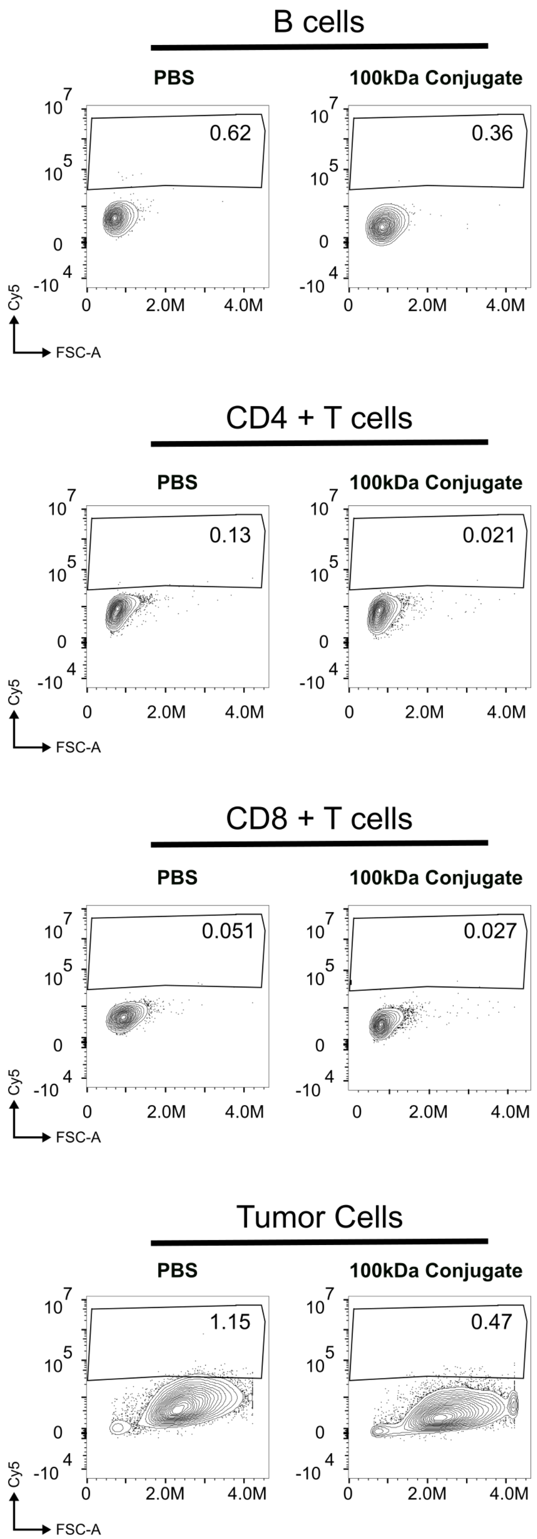

Figure S29: Flow cytometry gating for Cy5 uptake in other cell populations within the tumor

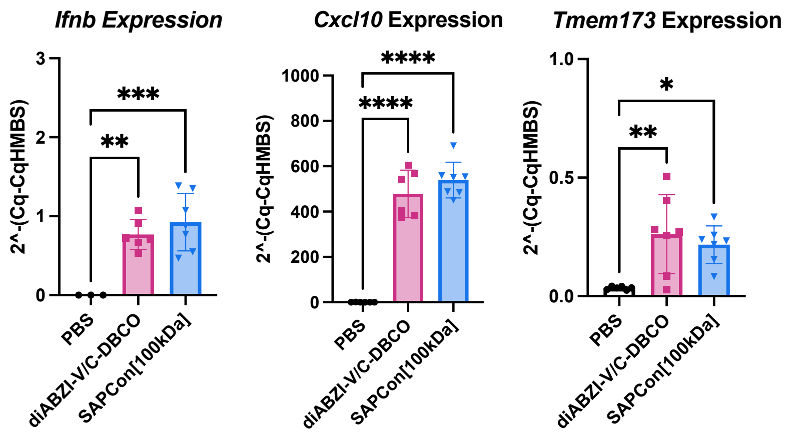

Figure S30: qRT-PCR analysis of liver tissue 6h following treatment of mice with PBS, diABZI-V/C-DBCO, or SAPCon[100kDa].

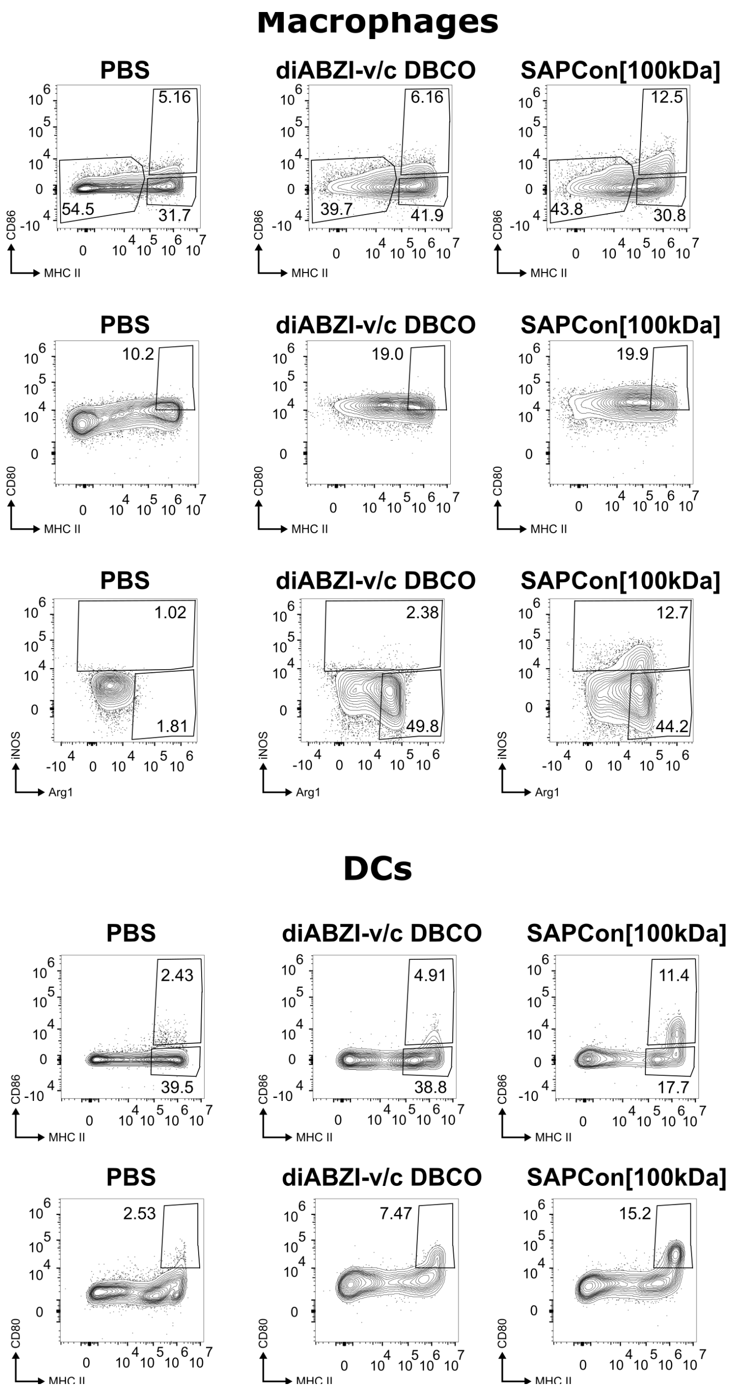

Figure S31: Gating scheme for macrophage and dendritic cell activation within the tumor

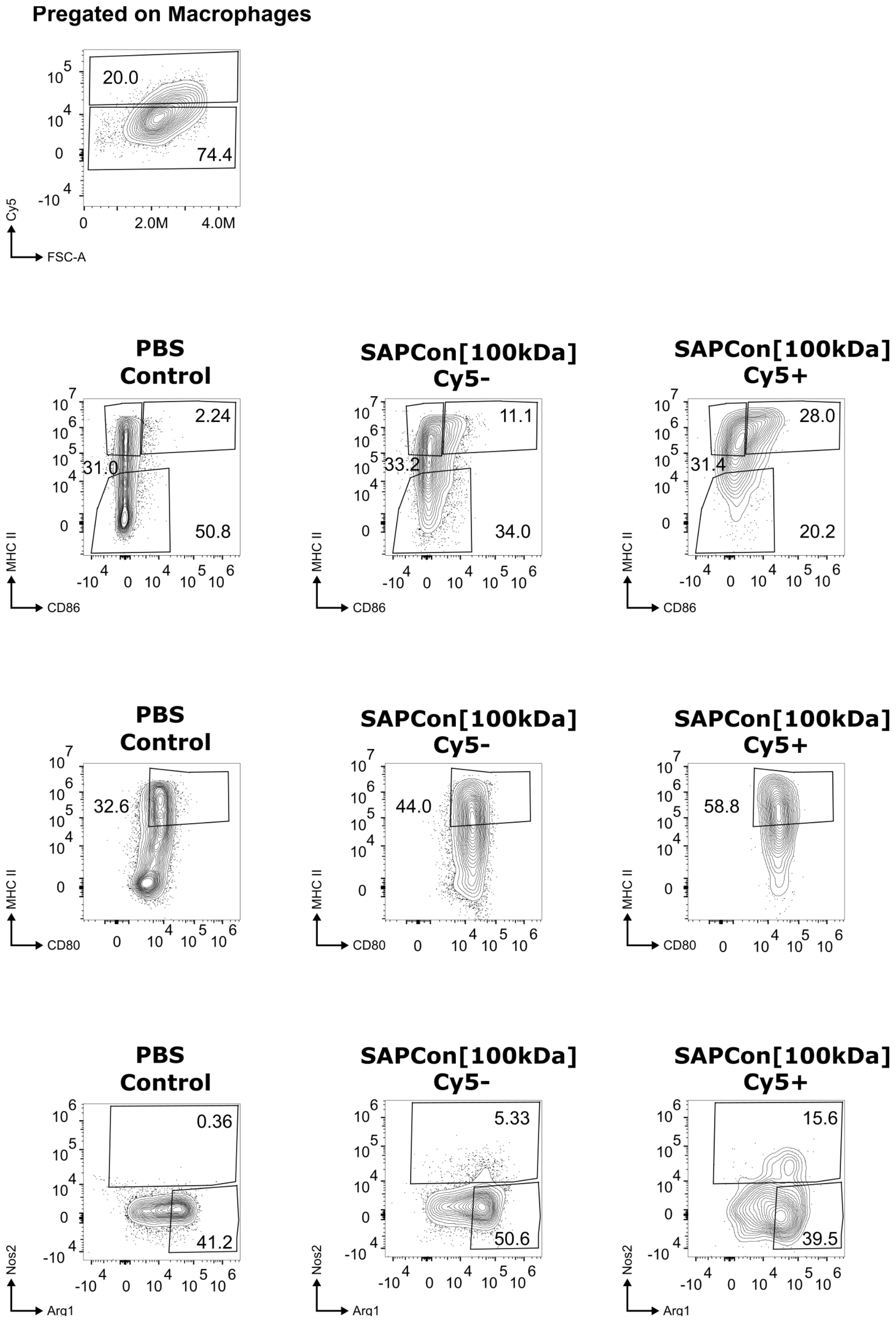

Figure S32: Gating scheme for Cy5 uptake and macrophage activation within the tumor

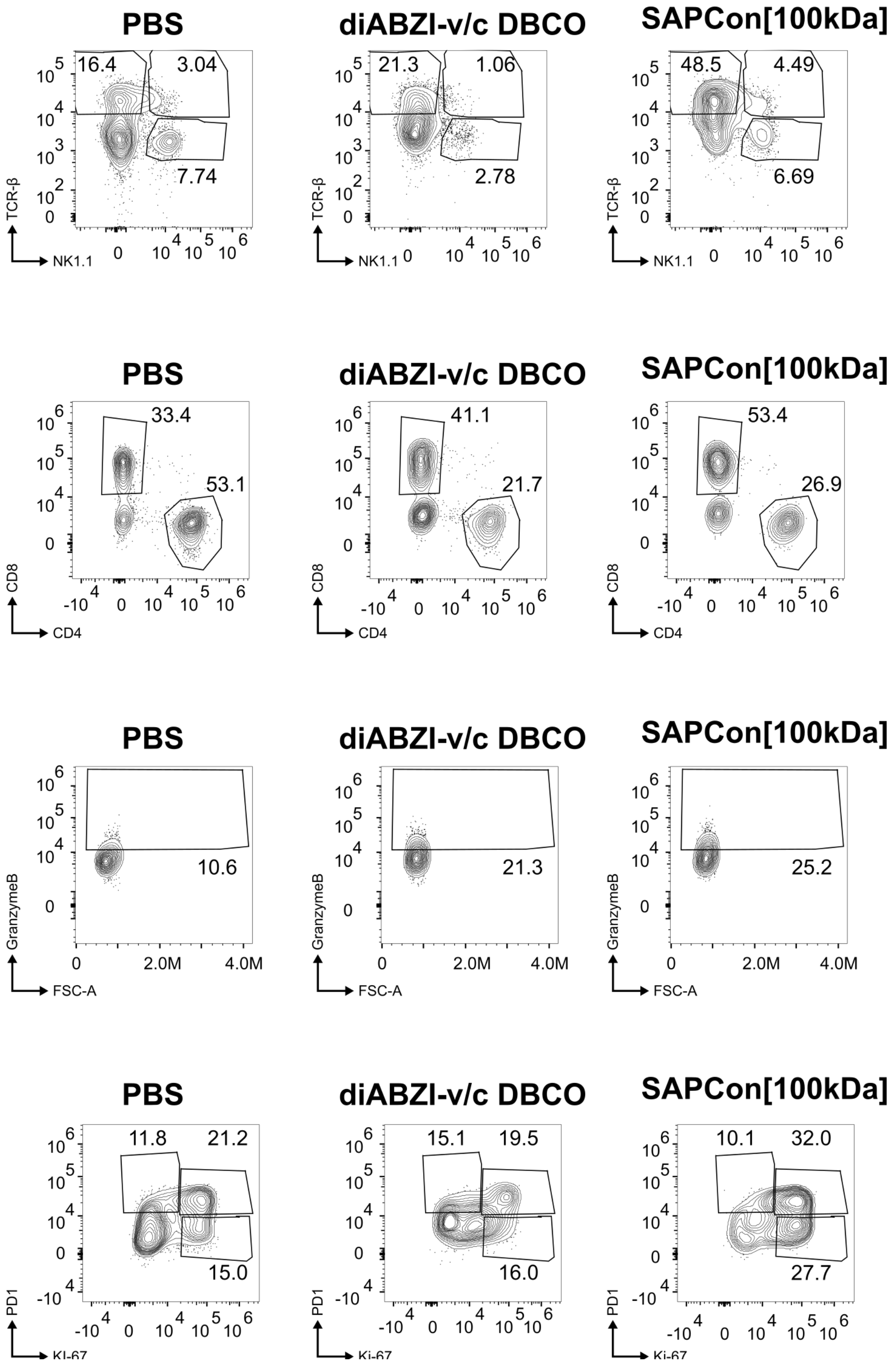

Figure S33: Gating scheme for T cell identification within the tumor

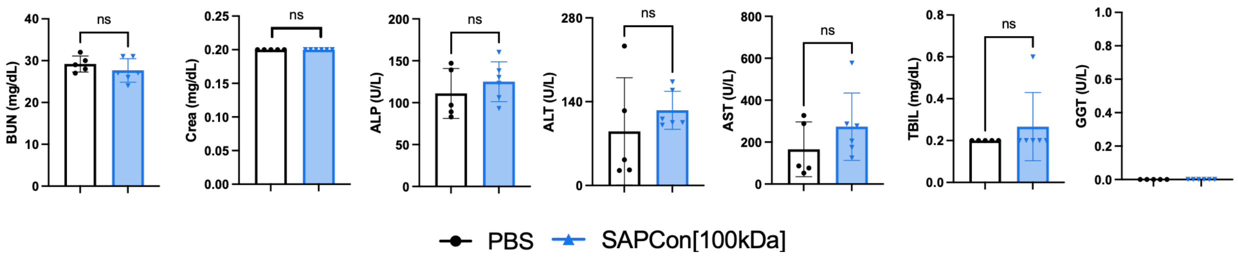

Figure S34: Blood toxicity marker analysis post treatment in healthy C57BL/6 mice (n=6)

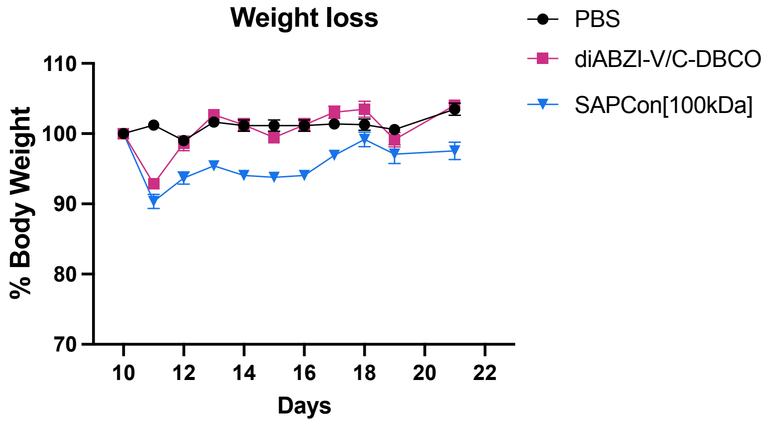

Figure S35: Evaluation of mouse body weight in response to treatment with SAPCon[100kDa] and diABZI-V/C-DBCO as described in Figure 6A.

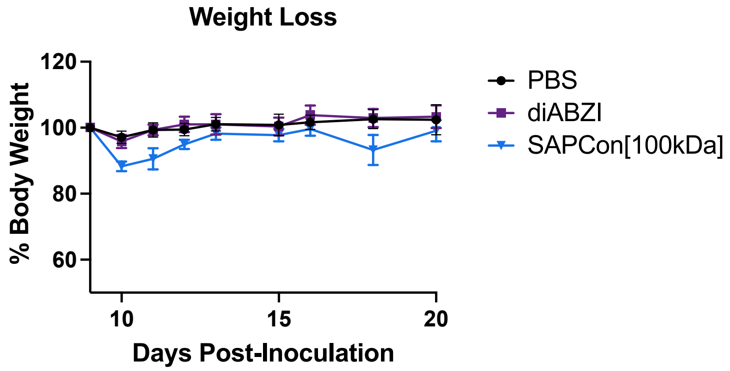

Figure S36: Evaluation of mouse body weight in response to treatment with SAPCon[100kDa] and diABZI (Compound 3) as described in Figure 6F.

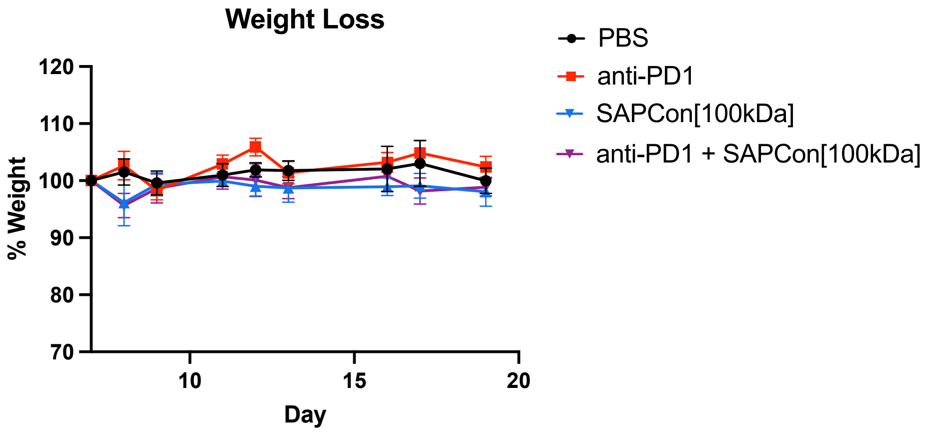

Figure S37: Evaluation of mouse body weight in response to treatment with SAPCon[100kDa], anti-PD-1 antibody, or SAPCon[100kDa] + anti-PD1 antibody as described in Figure 6J.
